# Fungal Lysine Deacetylases in Virulence, Resistance, and Production of Small Bioactive Compounds

**DOI:** 10.3390/genes12101470

**Published:** 2021-09-23

**Authors:** Ingo Bauer, Stefan Graessle

**Affiliations:** Institute of Molecular Biology, Biocenter, Medical University of Innsbruck, 6020 Innsbruck, Austria; ingo.bauer@i-med.ac.at

**Keywords:** lysine deacetylase (KDAC), histone deacetylase (HDAC), filamentous fungi, virulence, fungal disease, invasive pulmonary aspergillosis (IPA), pharmacological inhibition, histone deacetylase inhibitor (HDACI), secondary metabolism (SM), natural products

## Abstract

The growing number of immunocompromised patients begs for efficient therapy strategies against invasive fungal infections. As conventional antifungal treatment is increasingly hampered by resistance to commonly used antifungals, development of novel therapy regimens is required. On the other hand, numerous fungal species are industrially exploited as cell factories of enzymes and chemicals or as producers of medically relevant pharmaceuticals. Consequently, there is immense interest in tapping the almost inexhaustible fungal portfolio of natural products for potential medical and industrial applications. Both the pathogenicity and production of those small metabolites are significantly dependent on the acetylation status of distinct regulatory proteins. Thus, classical lysine deacetylases (KDACs) are crucial virulence determinants and important regulators of natural products of fungi. In this review, we present an overview of the members of classical KDACs and their complexes in filamentous fungi. Further, we discuss the impact of the genetic manipulation of KDACs on the pathogenicity and production of bioactive molecules. Special consideration is given to inhibitors of these enzymes and their role as potential new antifungals and emerging tools for the discovery of novel pharmaceutical drugs and antibiotics in fungal producer strains.

## 1. Introduction

The physiologically diverse fungal kingdom plays an indispensable ecological role in the recycling of organic material and as symbionts of bacteria, plants, and animals on land or in aqueous habitats. For decades, fungal model systems have been used to study basic cellular processes with results applicable to higher eukaryotes, including humans. Moreover, some fungal species exert direct benefit as relevant factors in industrial processes, such as the production or bioconversion of organic compounds and the fermentation of food, or are exploited in the production of medically important secondary metabolites (SMs) [1]. Others, however, are acting as parasites or dreaded pathogens of plants and animals [2,3,4,5]. In humans, they are responsible for superficial, invasive, and systemic infections. In superficial infections, skin and mucous membranes are frequently affected by dermatophytes or yeasts. They occur mainly in healthy individuals and are, although debilitating and painful, commonly not life-threatening and easy to diagnose and to cure [6]. In contrast, invasive or systemic infections most frequently concern immunocompromised patients and pose a serious problem due to high mortality rates [7,8]. *Candida*, *Cryptococcus*, and *Aspergillus* are the main genera causing these mycotic diseases [9,10]. For instance, aspergillosis infections by *Aspergillus fumigatus* affect more than 300,000 patients each year with mortality rates of 30–95% [11].

Considering the poor prognosis for patients suffering from systemic infections in advanced stages and the fact that available drugs might have severe side effects, development of novel and more efficient therapies is a matter of urgency [12,13]. This is even more important as fungal pathogens are increasingly developing resistances to conventional therapeutics [14,15]. Nevertheless, most novel compounds in the antifungal pipeline are derivatives of already-used azoles, echinocandins, and polyenes, which are interfering with ergosterol biosynthesis, membrane integrity, and cell wall synthesis, respectively [16]. Alternative treatment strategies need to meet two main requirements: (i) their targets must be crucial for the vitality of the pathogen in vivo (or have to be at least important for the expression of critical virulence factors), and (ii) concentrations of the antifungals required to block those target molecules have to be well tolerated by the infected host. When it was shown a decade ago that a class-1-type lysine deacetylase (KDAC) is essential for the vitality of several filamentous fungi [17], this group of enzymes came into focus as possible target molecules in antifungal therapy. The fact that effective natural and synthetic KDAC inhibitors (KDACIs) were already known, some of which were successfully approved as drugs against certain other diseases [18], spurred the idea to consider these enzymes as vulnerable fungal targets and KDACIs as promising antifungal agents. To what extent KDACs might indeed affect the virulence of pathogenic fungi and what insights their depletion has yielded so far regarding fungal virulence is subject of one part of this review.

Among the factors contributing to fungal virulence are small bioactive molecules acting as mycotoxins. When spoiled food is consumed, these toxins have a variety of detrimental effects, ranging from poisoning and allergic reactions to triggering of cancer development [19]. On the other hand, the range of secondary metabolites (SMs) includes pharmacologically exploited compounds, such as antibiotic, immunosuppressive, anticholesterolemic, and anticancer agents; cholecystokinin and neurokinin antagonists; ion channel ligands; and even antifungal drugs [20]. Therefore, a huge number of lives are saved every year due to the administration of (modified) fungal products. Chromatin-based regulation of biosynthetic gene clusters is a decisive factor for SM production [21]. In the last decade, the impact of chromatin modifications for SM production was confirmed in many fungal species (for review, see [22,23,24,25,26]), and particularly the acetylation status of lysine residues on core histones and transcription factors (TFs) was found to play a central role [27,28]. The impact of classical KDACs for fungal SM production is another subject of this review.

## 2. The Fungal KDAC Repertoire: More Than Just the Number of Enzymes

**Figure 1:**
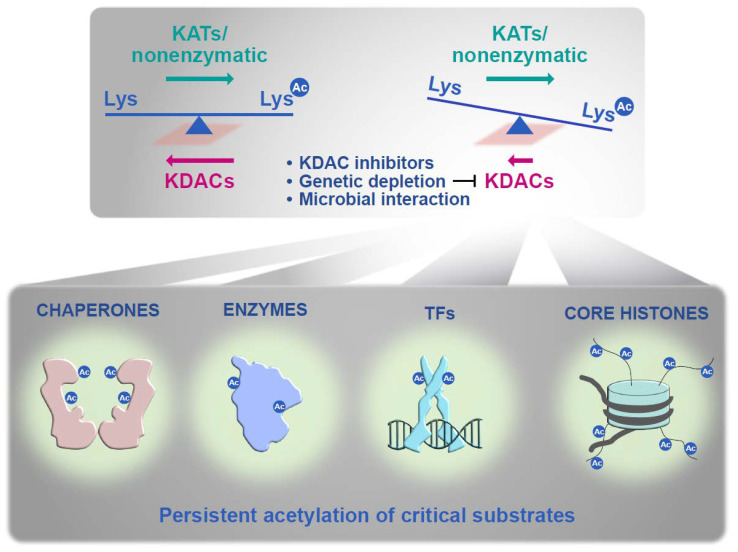
Maintenance of a delicate acetylation equilibrium. Disturbance of lysine deacetylase (KDAC) activity results in a destabilization towards persistent acetylation of critical substrates.

In this review article, we will focus on the Zn^2+^-dependent “classical” KDACs, which, together with lysine acetyltransferases and NAD^+^-dependent sirtuins, are maintaining a balanced acetylation status of their target proteins (Figure 1) [29]. Classification of KDACs is based on their homology to baker’s yeast Rpd3 and Hda1 as class 1 and class 2 enzymes, respectively. The standard fungal KDAC repertoire comprises at least two enzymes of each class, which are orthologs of yeast Rpd3 and Hos2 for class 1 and Hda1 and Hos3 for class 2 [21]. Nevertheless, there are examples of fungi containing additional paralogs of the Rpd3-type enzyme (e.g., *Aspergillus flavus*, *Candida* *albicans*, *Ustilago maydis*, and *Cryptococcus neoformans*) [30,31,32,33,34]. Most KDACs are considered members of multisubunit protein complexes, and whereas class 1 enzymes mainly reside in the nucleus, class 2 enzymes frequently shuttle between the cytoplasm and nucleus and are therefore usually evenly distributed throughout the hyphae [21]. Table S1 gives a comparative overview of the KDACs of selected fungal species discussed in this review, presenting their names and accession numbers.

The composition of fungal KDAC complexes has been elucidated mainly in the model yeasts *Saccharomyces cerevisiae* [35,36,37,38,39,40,41,42,43] and *Schizosaccharomyces pombe* [44,45,46,47]. Most studies focused on the composition of class 1 KDAC complexes. Each model yeast contains one Hos2 complex. This so-called Set3C complex harbors the lysine methyltransferase Set3 and in baker’s yeast also the sirtuin Hst1 [38,48]. In line with a corepressor function generally attributed to KDACs, disruption of Set3C led to premature induction of meiosis, accompanied by derepression of the corresponding genes in *S. cerevisiae* [38]. Nevertheless, the Set3C KDAC Hos2 and its *Cochliobolus* ortholog Hdc1 were the first KDACs shown to be directly involved in gene activation [49,50]. In budding yeast, Set3C binds to coding regions of actively transcribed genes and is required for full transcription [50]. However, it also reduces histone acetylation levels near 5’ ends of genes, where it is recruited by H3K4 dimethylation catalyzed by COMPASS (Set1) and the following H2B SUMOylation [51,52], thereby suppressing spurious transcription [52].

At least three complexes each of Rpd3 and Clr6—the *S. pombe* Rpd3 homolog—exist in budding and fission yeast, respectively. Apart from the stress-responsive *S. cerevisiae* Rpd3µ complex [53], these complexes, termed Rpd3L, Rpd3S, Clr6I, Clr6I’, and Clr6II, belong to the highly conserved Sin3 family [54]. Sin3 complexes consist of a trimeric core composed of Rpd3, Sin3, and Ume1 in *S. cerevisiae* or their respective orthologs in other fungi.

Rpd3L mediates gene repression at promoter regions, where it is recruited by sequence-specific transcription factors [40,55,56,57] and is crucial for transcriptional repression memory [58]. Rpd3S locates to promoter regions and to open reading frames in the wake of elongating RNA polymerase II to suppress transcription from cryptic promoters [39,41,59]. Equivalents of Rpd3L and Rpd3S complexes, Clr6I and II, are also found in fission yeast and similarly are implicated in the regulation of chromatin structure and transcription [45,47,60]. In contrast to Rpd3, however, *S. pombe* Clr6 is essential [44], and no homolog of the Rpd3µ complex has been reported in fission yeast so far.

Recently, an increasing number of biochemical studies addressing KDAC complexes have been published also in other fungi, including important (plant) pathogens, such as *C. albicans*, *Magnaporthe oryzae*, and *Fusarium pseudograminearum* [61,62,63,64].

The composition of the Hos2 containing the Set3C complex has been elucidated in *C. albicans* [61]. The *C. albicans* Set3C complex represses the virulence-critical yeast to hypha transition by precisely modulating the expression timing of critical factors governing morphogenesis [61,65]. Further, Set3C is mandatory for biofilm formation, which is a hallmark of antifungal resistance [62]. The members of the Set3C corresponding TIG1 complex from *M. oryzae* have also been implicated in virulence [63]. Although Hos2 and Hda1 have been shown to be part of an Rpd3L expanded complex in *S. cerevisiae* and *S. pombe* [66], the *Aspergillus nidulans* Hos2 ortholog HosA was never identified by tandem affinity purifications (TAPs) with RpdA as bait [67]. RpdA TAP in *A. nidulans,* however, led to the identification of a new complex termed RcLS2F [67]. The RcLS2F complex represses genes in a KDAC activity-dependent manner and is essential for sexual reproduction [67]. This complex consists of the conserved Sin3–Ume1/Prw1/Rpd3 core that is also part of the RpdAL and RpdAS complexes, which have counterparts in *S. cerevisiae* and *S. pombe* as well [67]. The defining members are ScrC and FscA; the former has already been described as extragenic suppressor of *crzA* [68]. CrzA is a transcription factor acting downstream of calcineurin signaling (see below) with a putative link to virulence and drug resistance (reviewed in [69]). Rpd3-type complexes have also been described in *C. albicans*. Interestingly, whereas Rpd3 and Rpd31 both interact with defining members of the large complex, Rpd31 was exclusively found in the small complex, probably explaining the opposing roles of the *Candida* Rpd3 paralogs in white-opaque switching [31]. Further, Rpd31 has also been demonstrated to directly interact with Ssn6, a critical factor for filamentous growth and virulence [70].

Fungal class 2 KDAC complexes have been less extensively studied. The composition of the Hda1 complex consisting of an Hda1 homodimer and an Hda2–Hda3 heterodimer has been unraveled in *S. cerevisiae* [35,43,71]. In addition to the Hda1 subunit harboring the catalytic domain, Hda2 and Hda3 have been shown to be mandatory for in vitro catalytic activity and reporter gene repression [71]. However, no orthologs for Hda2 or Hda3 could be identified outside *Saccharomycotina* by sequence analysis. An Hda1-ortholog-containing complex was identified also in *S. pombe*. The corresponding SHREC complex consists of the KDAC Clr3, the chromatin remodeling factor Mit1, and the less conserved partners Clr1 and Clr2 [46]. The *A. nidulans* Hda1 ortholog HdaA also elutes as a multimeric complex in size exclusion chromatography [72]; its composition, however, has not been elucidated yet.

## 3. The Role of Lysine Deacetylases in Fungal Pathogenicity and as Drug Targets for Novel Antifungal Agents 

Several fungal species are causative agents of serious human infections or are causing losses of billions of dollars by acting as pests of crops or as putrefactive agents of food. Upon infection, enormous adaptive power is needed by a fungal pathogen to overcome the host immune response. This is reflected by the massive change in the transcriptional program observed in a variety of pathogens during infection (e.g., [73,74]). Shaping the chromatin landscape, KDACs must be regarded as critical factors for such drastic reorganization of gene expression. Their influence either on the regulation of critical virulence and resistence factors or on fungal vitality itself has attracted considerable attention for the role of these enzymes as potential drug targets in antifungal therapies. On the one hand, there is ever-increasing evidence of attenuated virulence of fungal pathogens when their KDAC function is disrupted chemically or genetically. On the other hand, inhibition of KDAC activity significantly increases the efficacy of established antifungal drug therapies. Both aspects will be thoroughly discussed in the following chapters.

The first report on a fungal KDAC as a virulence determinant was on the class 1 enzyme Hdc1 in the plant pathogen *Cochliobolus carbonum* [49]. Deletion of *HDC1* resulted in significant virulence attenuation, which was attributed to a lack of expression of plant-cell-wall degrading enzymes [49]. In the meantime, virulence defects of KDAC deletion strains have been reported for plant and insect pathogens throughout all fungal phyla, such as *Botrytis cinerea* [64], *Fusarium fujikuroi* [75], *Fusarium graminearum* [76], *F. pseudograminearum* [77], *M. oryzae* [63,78,79,80,81,82], *U. maydis* [83], and *Beauveria bassiana* [84,85]. Most of these studies focused on loss of function mutations (i.e., gene knockouts), and many studies identified Hos2 orthologous enzymes as being critical for virulence. This, however, may be owing to the fact that, except for *F. graminearum* [77], attempts to delete genes encoding RpdA-type enzymes failed for several ascomycete mold pathogens, such as *A. fumigatus*, *A. flavus*, *B. cinerea*, *F. fujikuroi*, and *M. oryzae* [30,75,78,86,87]. Therefore, expression strategies via tunable promoters have been implemented to analyze RpdA-type enzymes [17,88]. Overexpression (OE) of *B. cinerea* Rpd3 caused histone hypoacetylation and defects in growth, sporulation, and virulence [87]. A virulence defect due to Rpd3 OE has also been reported for *M. oryzae* [82]. In contrast to *B. cinerea*, however, OE of Rpd3 resulted in increased sporulation in *M. oryzae* [82]. Interestingly, OE of the Rpd3 ortholog RpdA did not result in such phenotypes in *A. nidulans* or *A. fumigatus*; the very same growth phenotypes, however, were associated with depletion of RpdA in these fungi [17,89]. The observed differences illustrate the evolutionary distance between different fungal phyla and clearly demonstrate the importance of the proper balance of acetylation and deacetylation activities.

### 3.1. KDACs as Virulence Determinants of Human Fungal Pathogens

Three fungal genera are of outstanding importance as systemic human pathogens, mainly infecting immunocompromised patients: *Candida*, *Cryptococcus*, and *Aspergillus*. The difficulties in diagnosis and the development of resistance upon treatment result in high mortality of invasive fungal infection [90,91]. A role for KDACs as virulence determinants has been reported for all three genera.

The class 1 KDAC Rpd31, one of two Rpd3 paralogs, has been shown to be crucial for the virulence of C. *albicans* in a mouse invasive infection model [70]. Although no other *Candida* KDACs have been studied with respect to pathogenicity so far, indirect evidence implicates Hos2 as another relevant factor. Deletion of the Hos2 interaction partner Set3, for instance, resulted in significant virulence attenuation, and Hos2 and Set3, as members of the same complex, phenocopy each other in many aspects [65].

The genome of the basidiomycete yeast *C. neoformans* harbors six KDAC genes. Whereas one clear ortholog can be assigned each to Hos2, Hda1, and Hos3, four Rpd3 paralogs (Rpd301–304) are present, with Rpd304 being most closely related to *S. cerevisiae* Rpd3. The first evidence for a role of *Cryptococcus* KDACs in virulence emerged in a reverse genetic screen for virulence determinants [92]. Pools of selected genetically barcoded mutant strains, originating from signature-tagged mutagenesis (STM, [93]), were used for simultaneous pulmonary mouse infection. Upon appearance of clear signs of morbidity, the animals were sacrificed, and the relative abundance of each mutant tag was compared with that prior to infection. The resulting STM scores, which are a measure for survival of the respective mutant in vivo, could be well correlated to the actual virulence of selected genes. In this screen, the class 1 KDACs Hos2 and Rpd304 were among those genes with the lowest in vivo survival rates, indicating significant impairment of infectivity. Mutations of Hos2 and other members of the corresponding Set3 complex exhibited enlarged capsules and melanization defects, indicating increased immune evasion and decreased fitness, respectively. In contrast to Hos2, however, Rpd304 deletion did not show any phenotypes in addition to the STM score reduction. Two other Rpd3 paralogs were also tested but did not show significant changes of their STM scores; other KDAC mutants were not included in the library [92]. Nevertheless, a *C. neoformans HDA1* deletion mutant turned out to be avirulent in a murine inhalation model [94]. In line with this, Δ*hda1* showed defects for critical virulence-associated phenotypes, such as capsule formation, melanization, protease production, and resistance to various stressors [94]. In a comprehensive comparative study on the role of lysine acetylation in the most prevalent human pathogens, Ding and colleagues reported generation of *Cryptococcus* strains individually deleted of all six KDAC genes and assessed their performance in a mouse model for invasive infection. They observed a pronounced impairment of virulence upon deletion of Hos2 and Hda1 [34]. Surprisingly and in contrast to results from other reports [92], the Rpd304 mutant did not show strong deficits. However, its virulence was attenuated, as was also the case for Rpd302 and Rpd303 [92]. It is tempting to speculate that Rpd3 paralog functions may be at least partly redundant and that deletion of multiple paralogs might have led to a significant attenuation. The authors themselves attributed the apparent discrepancies to rapid microevolution of this fungus [34].

In contrast to the aforementioned pathogenic fungi, the *A. fumigatus* Rpd3 ortholog, RpdA, is essential for the life of the fungus under axenic growth conditions [86]. Moreover, RpdA was recently confirmed as a critical virulence determinant of *A. fumigatus* in a mouse model for invasive pulmonary aspergillosis [89]. To achieve rpdA knockdown in vivo, the gene was controlled by the *P. chrysogenum xylP* promoter (*rpdA^xylP^*), and therefore, *rpdA* expression was made tunable by xylose. Virulence was assessed in a neutropenic murine model for invasive aspergillosis, and xylose was administered in the drinking water for the induction of *rpdA*. While the survival curves of xylose-supplemented mice infected with an *rpdA^xylP^* mutant and wild type were similar, the uninduced *rpdA^xylP^* strain remained avirulent. Therefore, RpdA must be regarded as a virulence determinant of *A. fumigatus*. Furthermore, no redundancy of KDAC functions was observed in this respect. By contrast and unlike *Cryptococcus* Hda1, *A. fumigatus* HdaA appeared not to be involved in virulence mechanisms in a mouse model [95]. 

In addition to the already-mentioned genera, species of the genus *Fusarium* showed dependency of KDAC function for full virulence on host plants [64,75,76,77,96]. This might also be relevant in human infection due to an increasing number of reports of life-threatening disease caused by fungi of this genus [97]. Studies reporting the virulence defects of fungal KDAC mutants are summarized in Table 1.

### 3.2. Putative KDAC Targets Involved in Virulence and Antifungal Resistance

**Figure 2:**
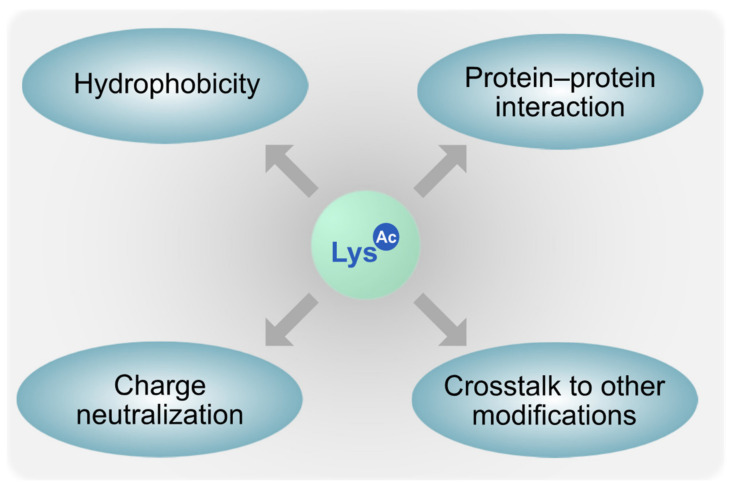
Possible biochemical consequences of lysine acetylation.

The fact that many KDAC mutants studied so far exhibit an obvious virulence phenotype raises the question on the cause(s) for these observations. Given the enzymatic nature of KDACs, it is very likely that erroneous acetylation of distinct cellular target proteins might be the main reason. The N-ε-acetylation of lysines leads to charge neutralization and increased hydrophobicity and affects protein–protein interaction (PPI) and crosstalk with other post-translational modifications (Figure 2, reviewed in [98]).

This hypothesis, however, is raising several questions:

- Is the virulence phenotype a result of changed gene expression patterns caused by altered chromatin regulation (i.e., hyperacetylation of histone tails at specific genomic loci like those coding for virulence factors or other proteins critical for fungal fitness)?
- Are (hyper)acetylated transcription factors of such critical genes responsible for the altered virulence behavior?
- Is a single or are multiple (hyper)acetylated protein(s) contributing to attenuated virulence?
- Is it a multifactorial process (i.e., a combination of the aforementioned points)?

In general, three features are essential for a human fungal pathogen to successfully establish infection: thermotolerance, ability to grow through host tissue, and lysis and absorption of this tissue [100]. A critical virulence factor, therefore, should affect one of those properties either directly or indirectly. As KDACs are important players in chromatin-level gene regulation via the deacetylation of histone tails and other chromatin proteins, it is plausible that certain virulence defects in KDAC mutants can be attributed to misregulation of gene expression caused by histone hyperacetylation. Nevertheless, it is also conceivable that disordered gene regulation on a global scale, as has been shown for fungal KDAC mutants and other factors involved in chromatin regulation [89,94,101,102,103], results in a general reduction of fitness. This might even not be recognized under standard laboratory growth conditions, but can be critical for survival and thriving in the host niche, where various stressors trigger changes in fungal gene expression via stress response pathways (e.g., [73,74]). Besides those global effects on transcription, a more direct impact on the acetylation of nonhistone targets might affect virulence. Indeed, thousands of acetylated nonhistone proteins have been identified in eukaryotes and prokaryotes (reviewed in [104]). Reflecting the ancient origin of protein lysine acetylation in the prokaryotic domain, proteins identified in such studies belong to highly conserved cellular processes such as translation and metabolism [105,106,107,108]. As has been discussed recently, however, many of the identified acetylation sites probably are present in very low stoichiometry and therefore might represent signs of cellular stress rather than having a direct regulatory impact [98]. Within the fungal kingdom, a vast number of acetyl-lysine proteins were also detected in pathogens such as *A. fumigatus*, *C. albicans*, *C. neoformans* [34,109], *Trichophyton rubrum* [110], *F. graminearum* [96], *B. cinerea* [111], and *B. bassiana* [84]. Notably, comparison of the acetylomes of the three opportunistic fungal pathogens *C. albicans*, *C. neoformans*, and *A. fumigatus* revealed that around 40% of all acetylated proteins present in these species could be assigned to infection [34]. Furthermore, there are examples of proteins whose acetylation has been shown to be critical for the survival in the host. One of the best-studied examples is the highly conserved heat shock protein 90 (Hsp90), whose genetic depletion led to virulence attenuation in C. *albicans* and *A. fumigatus* [112,113]. Early studies revealed that pharmacological KDAC inhibition leads to hyperacetylation of Hsp90 and interferes with its ability to bind client proteins and cochaperones [114,115]. This was later linked to a highly conserved lysine residue in the middle domain of the chaperone [116]. Acetylation-mimicking mutations of this residue (K271A) together with a lysine in the N-terminal ATPase domain (K27A) resulted in significant attenuation of virulence in a murine IPA model [88] accompanied by decreased inflammation and hyphal invasion of the host tissue [88]. These observations indicate that active deacetylation of Hsp90 is mandatory to establish the proper acetylation equilibrium required for efficient infection and that Hsp90 might indeed be one of the KDAC substrates critical in virulence. Whereas deletion of both *HDA1* and *RPD3* induced acetylation of those lysine residues in *Candida* [117], KDAC(s) responsible for Hsp90 deacetylation in *A. fumigatus* have not been identified yet. Furthermore, Hda1 and Rpd3 are not the only KDAC-deacetylating Hsp90. Indeed, the physical interaction of Hos2 and Hsp90, as well as the contribution of all *Candida* KDACs to drug resistance, has been shown [118]. Interestingly, acetylation of K271 but not K27 could be induced by pharmacological KDAC inhibition in *Aspergillus* [88]. Despite these clear effects, however, Hsp90 is not a suitable antifungal target for clinical use owing to its extremely high conservation and the concomitant severe side effects.

Several acetylated proteins from *C. neoformans* have also been tested by mutation analyses, indicating the importance of this modification for virulence. However, depending on the protein assessed, positive and negative effects of acetylation-mimicking mutations were observed [34]. For instance, translation elongation factor 1 (Tef1), another essential and highly conserved protein, also seems to be involved in the virulence defects of KDAC mutants of *C. neoformans*. Tef1 directly interacts with class 1 and class 2 KDACs, and deletion of these enzymes clearly led to hyperacetylation of Tef1. Furthermore, the acetylation-mimicking mutation (K to Q) of three fungal-conserved Tef1 lysine residues (K36, K41, and K217) resulted in severe growth reduction [34].

### 3.3. KDACs as Modulators of Fungal Drug Resistance

Sublethal doses of antifungal compounds trigger a stress response that might result in antifungal resistance. The main routes of resistance development are reduction of drug-binding affinities via point mutations, increased expression of their targets, and increased intracellular clearance of the antifungal drug by the upregulation of efflux pumps (see [119,120,121] for review). Since KDACs are important regulators of chromatin structure and thus of global gene expression patterns, they have also been considered as targets to overcome secondary resistance in *Candida* and other fungal pathogens [122,123]. In the first study addressing the effect of KDAC inhibition on antifungal sensitivity in *C. albicans*, Smith and Edlind observed clear synergism in growth inhibition by the KDAC inhibitor trichostatin A (TSA) and azoles but not polyenes or echinocandins [122]. They also observed that KDAC inhibition prevented the upregulation of ergosterol biosynthetic enzymes and drug exporters [122]. Later, this initial observation was confirmed and functionally linked to Hsp90 acetylation [117]. Hsp90 was shown to potentiate the rapid evolution of drug resistance in *Candida* and *Aspergillus* [124]. Several typically acquired mutations leading to drug resistance were found to be dependent on functional Hsp90 and its client protein calcineurin [124]. TSA treatment mimicked the inhibition of Hsp90 by geldanamycin, and both abolished the development of azole resistance [117]. Notably, this observation was not the result of Hsp90 depletion, since Hsp90 transcript and protein levels remained unchanged upon TSA treatment [117]. Interestingly, in contrast to previous observations [122], expression of key enzymes involved in antifungal resistance was also not changed by KDAC inhibition in this study [117].

Another exciting fact linking Hsp90, deacetylation, and antifungal resistance is the observation that Snt1, a scaffold subunit of the Hos2 KDAC complex Set3C, can be transformed into the prion [*ESI*^+^] [125]. This switch triggered by phosphorylation results in increased Set3C recruitment to subtelomeric chromatin, where it drives the expression of numerous stress-adaptive genes [125]. Indeed, [*ESI*^+^] heritably conferred increased resistance to fluconazole [125]. Importantly, [*ESI*^+^] was eliminated by even transient inhibition of Hsp90 [125]. Given that Hsp90 function is dependent on its deacetylation, it is likely that KDAC inhibition also results in a loss of the [*ESI*^+^] prion. However, this idea remains to be tested.

Lamoth and colleagues have extensively studied the role of K27 and K271 acetylation of Hsp90 in drug sensitivity in *A. fumigatus* [113]. Simultaneous mutation of these residues to acetyl-mimicking alanines led to significant impairment of Hsp90 function, resulting in hypersensitivity to azoles and echinocandins with loss of the paradoxical response [113]. In contrast to yeast, however, mutation of K271 alone was not sufficient to attenuate Hsp90 function [113,117]. On the other hand, K27A or deletion of K27 resulted in phenotypes almost reflecting double mutants [113]. The K27R mutant that mimics a constitutively unacetylated state caused mildly increased susceptibility against azoles but not against echinocandins, and also the paradoxical effect of caspofungin was not affected by this mutation [113]. Taken together, the role of acetylation in fungal pathogenicity and antifungal susceptibility tags KDACs as valuable novel targets in the treatment of fungal infections.

### 3.4. KDAC Inhibitors for the Treatment of Invasive Fungal Infections

One of the best characterized KDACIs, trichostatin A (TSA), was discovered as an antifungal compound in 1976 [126], long before its molecular function as KDACI was identified [127]. The observed deregulation of KDACs in several types of human disease, such as malignancies and cardiovascular and neurodegenerative disorders, increased the interest to explore the natural occurrence of KDACIs [128]. This led to the discovery of different types of KDACIs classified as hydroxamates, such as TSA, short-chain fatty acids, benzamides, and cyclic tetrapeptides; all are chelating the Zn^2+^ ion essential for enzymatic activity of classical KDACs (for review, see [129]). Currently, six KDACIs are approved against certain types of cancer, heart disease, and neurodegenerative disorders (e.g., [130,131,132]) by US or Chinese authorities, and several others are in clinical trials. Due to the essential role of mammalian KDACs, however, administration of KDACIs poses a risk of side effects. Furthermore, broad-spectrum KDACIs block the innate immune response [133], thus making the development of fungal-specific inhibitors desirable. The extraordinary conservation of the KDAC catalytic domain, however, complicates this task. Nevertheless, KDACIs able to discriminate class 1 and class 2 enzymes are available (e.g., the class 1 inhibitor entinostat) [134]. Regardless, extensive modeling or—preferably—experimentally solved structures of the respective fungal KDACs will be needed for the development of specific inhibitors.

In any case, TSA treatment inhibits the growth and germination of the ascomycete *A. fumigatus* [86,113], and in the basidiomycete pathogen *C. neoformans,* KDAC inhibition decreased virulence-relevant traits, such as capsule size, melanin formation, and ability to grow at 37 °C [135]. A fungal-specific KDACI, MGCD290, has been described to be specific for Hos2 in *Candida* species. MGCD290 acts synergistically with different azole drugs in yeast and molds and overcomes fks1/2-mediated resistance against echinocandin drugs in several *Candida* isolates [136,137]. Interestingly, despite synergistic growth inhibition by MGCD290 plus fluconazole in 6/10 *Aspergillus* isolates and its selectivity for Hos2 in yeast [136], deletion of the *HOS2* ortholog *hosA* in *A. nidulans* reduced azole susceptibility [103]. This indicates that HosA most likely is not the main target of MGCD290 in *Aspergillus*.

Efforts to identify fungal-specific KDAC inhibitors are still ongoing, although mainly focusing on the inhibition of yeasts. For example, a new drug simultaneously containing triazole and hydroxamic acid entities separated by a spacer inhibited the growth of azole-resistant C. *albicans* isolates [138]. Another example is the synthesis of a fungal-selective carboline KDACI that significantly reduced fungal growth in a mouse model [139]. In addition to the highly conserved catalytic domain, however, other specific domains of KDAC enzymes might be approachable. For instance, RpdA-type enzymes of filamentous ascomycetes share a remarkable C-terminal extension when compared with homologs from yeasts, plants, or mammals. This extension contains a conserved acidic patch critical for the enzymatic activity, proper nuclear localization, and even survival of *A. nidulans* [17,86]. In this sense, the C-terminal domain of RpdA-type enzymes might be a target for the development of an allosteric inhibitor.

### 3.5. Targeting Protein–Protein Interaction within KDAC Complexes: Another Possibility to Combat Fungal Infections?

One important aspect of the Zn^2+^-dependent KDAC proteins is their dependence on interacting proteins for full (catalytic) activity. The composition of these complexes increases the functional diversity of the four classical enzymes present in most of the fungal proteomes [21]. Importantly, in contrast to the enzymes themselves, most of the known fungal complex partners are not or poorly conserved between fungi and mammals. Thus, targeting the complex integrity or the function of KDAC interaction partners may allow for alternative ways of fungal-specific chemical intervention (i.e., by inhibition of essential PPI). For instance, disruption of RpdA-type complex components indeed led to decreased virulence in the plant pathogens *B. cinerea* and *M. oryzae* [64,78,82]. Despite the fact that pharmacological modulation of PPI is difficult to achieve, a number of studies reported the identification of small molecules for that purpose (as reviewed by [140]). Basically, three types of strategies can be used to interfere with PPI. In orthosteric inhibition, a small molecule directly competes with the interaction partners at the site of interaction (e.g., inhibition of acetyl-Lys binding by bromodomains via benzodiazepine compounds [141]). Allosteric inhibitors, on the other hand, act at sites not comprising the PPI interface, inducing either conformational or dynamic changes interfering with protein binding. A third type—the interfacial inhibition—describes the locking of a transition state by a small molecule. A prominent example for this type of inhibition is trapping of ADP-ribosylation factor (ARF) by brefeldin A [142]. Nevertheless, currently no such interference strategies are available for the inhibition of fungal KDAC complexes. For the development of such compounds, it will be important to consider potential compensation mechanisms of the organism that might compromise therapeutic success. For example, loss of the *S. pombe* Clr6I complex can be bypassed by the deletion of Clr6II-specific components [143]. Thus, targeting specific fungal KDAC complexes might be best achieved by targeting essential subunits. However, to exploit the disruption of complex-specific interactions as an antifungal strategy, further knowledge on the composition and function of KDAC complexes with respect to fungal virulence is needed.

## 4. Lysine Deacetylases as Regulators of Small Fungal Natural Products

Their importance as potential drugs, dreaded toxins, or putative virulence factors brought small natural products of fungi into the research focus of mycologists worldwide. The majority of these molecules, commonly termed secondary metabolites (SMs), are still unexplored—even in well-studied fungal genera such as *Aspergillus. A. nidulans*, for instance, encodes about 30 polyketide- and polyketide-like synthases (PKSs) and almost the same number of non-ribosomal and non-ribosomal-like peptide synthetases (NRPSs). The metabolites produced by many of those enzymes, however, are unknown [144]. The high quantity of putative metabolites in each single species, together with the huge number of fungi that are yet unstudied or unknown [145], might give an idea of the vast arsenal of useful bioactive natural products awaiting to be discovered. The significant lack of knowledge even in well-known fungi is reasoned by the fact that SMs are synthesized exclusively under very specific growth conditions or in distinct stages of fungal development. Moreover, many of those molecules are produced in very low amounts, which hampers detection. 

To identify novel fungal products, sophisticated strategies are thus required to stimulate their production under growth conditions in laboratories. This requires sound knowledge of the regulatory mechanisms of PKS- and NRPS-encoding sequences required for the SM synthesis. The fact that these genes are often clustered within the fungal genomes not only aids in their identification during genome mining but also facilitates their concerted regulation. In contrast to bacteria, however, no operons exist in fungi. Instead, higher-order regulatory mechanisms such as DNA methylation or chromatin modifications within gene cluster regions are common for a coordinated regulation of associated genes in eukaryotes [146]. Because the most prominent modification is the acetylation of N-terminal lysines of core histone proteins, preferentially those of H3 and H4, lysine acetyltransferases and deacetylases are also significantly involved in the regulation of SM production in filamentous fungi.

### 4.1. Impact of KDACs in the Regulation of SM Production: First Evidence

Two decades ago, soon after the first members of KDACs and KATs were identified in filamentous fungi [147,148], an initial link between KDACs and SMs was found when HC-toxin, an SM of *C. carbonum*, was identified as a putative inhibitor of plant KDACs and virulence factor, compromising defense mechanisms of the host plant [149]. The first evidence that KDACs might have a direct impact on SM production came in 2007 for *A. nidulans* [27]. It was shown for the first time that deletion mutants of the class-2-type KDAC HdaA show an early and enhanced induction of well-known SM genes of the antibiotic penicillin (PN) and the carcinogenic aflatoxin precursor sterigmatocystin. Interestingly, an upregulation of cryptic SMs was also observed in wild-type strains of fungal species, such as *Alternaria* or *Penicillium*, when trichostatin A (TSA) was added to the medium [27]. Because TSA inhibits classical KDAC activity, these results suggest that the acetylation status of histones and/or other regulatory factors have a significant impact on SM production. In contrast to HdaA deletion, however, TSA did not increase PN production in *A. nidulans*. This counterintuitive behavior was explained later when it was demonstrated that different types of KDACs might cause opposing regulatory effects on one and the same metabolite cluster (see below). The impact of HdaA on SM regulation was subsequently also confirmed for *A. fumigatus* HdaA [95]. Interestingly, however, qPCR results revealed that a few SM cluster genes of *A. fumigatus*, for instance, an NRPS of the gliotoxin cluster, were not up- but downregulated in an HdaA mutant strain. This indicates a possible dualistic function of this class-2-type KDAC, depending on the SM cluster investigated. Interestingly, such an unusual function of fungal KDACs was already demonstrated a few years earlier for the class-1-type enzyme Hdc1 of *C. carbonum* and even for *A. nidulans* HdaA on the regulation of non-SM genes [49,150].

### 4.2. Versatile Functions of KDACs in the Expression of SMs: First Functional Hypothesis

The obvious importance of KDAC activity for the regulation of SMs in filamentous fungi and data on the impact of the acetylation status in promoter regions of aflatoxin biosynthetic cluster genes in *Aspergillus parasiticus* [28] were the starting signal for investigations of KDAC mutants in other fungal species. These studies revealed exciting new insights into the multifaceted regulatory activities of different KDAC classes in *Aspergillus* spp. and *Fusarium* spp. and, in the recent past, also in several other genera.

In 2013, for instance, Studt et al. showed that the deletion of the classical KDACs Hda1 (class 2) and Hda2 (class 1) might have both repressing and enhancing effects on the production of distinct metabolites in *F. fujikuroi* [75], supporting the findings from *A. fumigatus* [95]. Interestingly, however, OE as well as depletion of Hda1 led to very similar effects on the regulation of the gibberellin and the bikaverin cluster. Two hypothetical models were proposed as possible explanation of this phenomenon. The idea of the first model was that deacetylation of a TF enables its binding to the corresponding promoter as prerequisite for the recruitment of a KAT complex. In balance with the opposing KDAC activity, this KAT complex promotes transcription by fine-tuning the acetylation status of the regulatory region. In case of Hda1 OE, KDAC activity overrules that of the KAT complex, leading to hypoacetylation of the promoter and consequently to a reduced transcription of the cluster genes. In case of KDAC deletion, however, the involved TF remains acetylated, preventing its access to the promoter and subsequent KAT binding. This again results in hypoacetylation of the promoter region and to repression of the corresponding gene cluster. In other words, both Hda1 depletion and Hda1 OE lead to reduced gibberellin production. 

In a second model, a KAT complex binds independently of a TF. In case of Hda1 deletion, the balance of the acetylation status of the corresponding chromatin structure is disturbed via hyperacetylation of H3K9, leading to open chromatin. This open structure, however, does not trigger increased promoter activity but enables the binding of a yet unknown repressor complex that prevents transcriptional activity of the bikaverin cluster [75]. 

These two basic models were subsequently complemented by a third one, in which the modification status of H3K27 (trimethylation or acetylation) adds to the regulation of the beauvericin cluster in *F. fujikuroi.* The activity of a still-unknown KAT resulting in hyperacetylation of K27 due to deleted Hda1 and hypomethylation by depleted Kmt6 thereby leads to an induction of the corresponding cluster genes [151].

Irrespective of the validity of all these models, it is widely accepted today that, in addition to histones, most classical KDACs can deacetylate TFs and other proteins [152], resulting in an unpredictable regulatory versatility of these enzymes. In *A. nidulans*, for instance, the class 1 enzyme HosA (a homolog of Hda2) can act as both repressor and inducer of SM clusters [103]. HosA deletion resulted in approximately 200 genes that were up- and more than twice as many that were downregulated, among them also several SM cluster genes. These data reconfirm the untypical role of KDACs as transcriptional inducers for some SM cluster genes. Regardless of whether induced or silenced, genes adjacent to the HosA-regulated gene clusters were hardly or not at all affected, demonstrating a high accuracy of these regulatory events [103]. One example of a cluster requiring HosA activity for its induction is the PN cluster. Consequently, PN production is completely disrupted in HosA delta strains. The fact that both KDACs, HdaA and HosA, have an impact on PN production in opposing directions led to the investigation of HdaA/HosA double mutants and revealed that enhanced PN production in HdaA deletion strains is overruled by the repressing effect of depleted HosA activity [103]. Retrospectively, this might explain why HdaA inhibition via pan-inhibitors such as TSA did not cause the expected increase in PN production in previous studies (see above).

### 4.3. Teamwork: KDACs Are Rarely Acting Alone

The cryptic role of HosA as a dominant inducer led to the assumption that yields of PN might be escalated by simple OE of this enzyme. This expectation, however, was not confirmed by subsequent experiments as even strong HosA OE did not affect PN production significantly [103]. Two explanations are conceivable for this fact. Either the amount of wild-type activity is already sufficient to deacetylate all targets responsible for SM induction and no further effect can be achieved by increased enzymatic activity, or OE of HosA alone does not result in an enhanced catalytic activity at all. Evidence for the latter hypothesis came from the above-discussed finding that almost all fungal KDACs are only active in large protein complexes. To the best of our knowledge, the sole exception of this rule is the yeast class 2 KDAC Hos3 and its orthologs in filamentous fungi. This KDAC type was found to exhibit a so-called “intrinsic” catalytic activity [72,153]. However, HosB seems to have only minor effects on SM production in filamentous fungi [27,75].

In the last two decades, several KDAC complexes were identified in yeasts and molds (see above). The hypothesis that a stoichiometrically balanced OE of KDACs and respective complex partners might be required to significantly increase catalytic activity therefore seems plausible. A concerted expression, however, would require profound knowledge about the composition of distinct KDAC complexes and their functions. Although corresponding investigations are currently in progress (e.g., [63,67,154]), successful increase of SM production by a balanced OE of relevant components of distinct complexes will remain a rather challenging strategy with an open outcome. The fact that a single KDAC might be part of different KDAC complexes with varying compositions further complicates this strategy.

### 4.4. Pan KDAC Inhibitors for Mining New SMs: Quick and Easy but Not Always Valid

In addition to enzymes that trigger SM production, a probably higher number of SM cluster genes are repressed by these enzymes. Thus, efficient depletion of KDAC activity can be a promising approach to successful genome mining for novel fungal products. As mentioned, a time-saving strategy to achieve this goal is the addition of KDAC inhibitors to the growth media. However, although quick and easy to perform, this strategy carries the risk of adverse effects induced by simultaneous inhibition of counteracting KDACs as described above for the enzymes HdaA (class 2) and HosA (class 1) in PN production. The application of class-specific inhibitors would be an alternative strategy, and such inhibitors actually exist. Compounds such as hydroxamic acid rocilinostat are exclusively targeting class 2 enzymes, whereas others, such as the benzamide entinostat or tacedinaline, are inhibiting class 1 KDACs only. The mere existence of such inhibitors implies that even minor differences within the otherwise highly conserved catalytic domains of class 1 and 2 KDACs can contribute to a significant class selectivity. Unfortunately, the repertoire of isoform-selective inhibitors is currently still small, and their effects on fungi are largely unexplored yet [155]. Consequently, most studies on fungi were performed with pan-inhibitors, such as TSA, suberoylanilide hydroxamic acid (SAHA, vorinostat), valproic acid (VPA), and sodium butyrate (NaB). Notwithstanding, these experiments led to interesting new findings about regulatory functions of KDACs. Almost one decade ago, the first comprehensive study was performed with various small chemical chromatin effectors that were evaluated regarding their impact on SM expression in *Aspergillus clavatus* [156]. This study revealed that NaB, VPA, and TSA have a strong impact on SM biosynthesis in a media-, time-, and target-dependent way. Subsequently, observations of other groups confirmed chemical epigenetic manipulations as a feasible strategy to trigger SM production in many species, such as *A. nidulans*, *Penicillium chrysogenum*, *Aspergillus calidoustus*, *Aspergillus westerdijkiae*, *Aspergillus versicolor*, and *Trichoderma harzianum* [157,158,159,160,161,162]. Although these publications uncovered dozens of interesting new metabolites, data obtained from different strains are heterogenous, and the complexity of the effects induced by the simultaneous inhibition of multiple KDACs makes the interpretation of the results sometimes difficult. 

### 4.5. Deletion of KDACs or Their Complex Partners for Mining New SMs: Reliable but Elaborate and Not Always Feasible

A more accurate possibility to deplete KDAC activity in SM producers is the generation of single or multiple KDAC gene deletion mutants. In contrast to the treatment with KDACIs, this strategy is more labor- and time-consuming and thus not applicable for fast screenings for novel SMs in a large number of fungal species. Nevertheless, deletion strategies are the first choice in well-established model fungi or industrial producer strains and, as already exemplarily mentioned above, has led to interesting knowledge about the impact of specific KDACs on the regulation of distinct SM gene clusters. In addition, more recent studies of deletion mutants also revealed first insights into how these enzymes might affect the production of fungal metabolites. One possible regulatory mechanism was recently published for HosA in *A. flavus* [30]. In this paper, authors show that HosA associates with the transcription factor SinA that recruits the SinA/HosA complex to specific promoter regions, triggering the transcription of the aflatoxin biosynthesis gene cluster. This implicates a decreased production of aflatoxin in both HosA and SinA delta strains. However, genomic clustering of associated metabolic genes is not at all a prerequisite for concurrent regulation via KDAC activity. Recent analyses of an *hdaA*-deleted *M. oryzae* strain revealed a concerted activation of three representative melanin genes that are dispersed in different chromosomes of the genome of this rice blast fungus [163]. Comprehensive deletion experiments in *Aspergillus niger* [164] involving eight coding sequences of putative sirtuins and classical KDACs under numerous stress conditions revealed that a Δ*hdaA* and a Δ*hosA* strain showed substantial dysregulation of SM expression. RNA-seq analysis in the Δ*hdaA* strain revealed approximately equal numbers of GO terms in up- or downregulated genes, yet more genes linked to the terms metabolic and biosynthetic processes were induced. In contrast, in Δ*hosA* most enriched GO terms were among downregulated genes, including many genes of nonribosomal peptide biosynthetic processes. These data reconfirm the results of previous studies in which HosA mainly positively affected SM production.

### 4.6. An Alternative Approach: Depletion of (Essential) KDACs via the Promoter Rundown Technique

Despite interesting insights gained through the deletion of distinct KDAC genes, this approach is not applicable for all types of fungal enzymes. Perhaps one of the most interesting KDACs, the yeast Rpd3 orthologous enzyme, is of vital importance for many filamentous fungi, and thus deletion mutants cannot be propagated as haploids [17]. For such essential enzymes, the application of a promoter rundown technique offers a practicable possibility to deplete catalytic activity for mining novel biomolecules. Exhaustion of catalytic activity is thereby achieved by replacing the promoter region of the respective KDAC gene with one that is conditionally repressible. The first KDAC that was depleted in a filamentous fungus using this technique was RpdA of *A. nidulans* [17]. To this end, two promoters were successfully employed: the alcohol dehydrogenase (*alcA*p)- and the xylanase (*xylP*p)-promoter. The latter was recently even successfully applied in vivo to repress *rpdA* in a non-neutropenic murine pulmonary aspergillosis model, confirming this enzyme as an important virulence factor of *A. fumigatus* (see above). Under in vitro conditions, survival of RpdA knockdown mutants can be ensured by moderate RpdA repression while simultaneously analyzing alterations of their biosynthetic repertoire on either the transcriptomic or the metabolomic level. Combining the *xylP*p rundown technique applied to RpdA in *A. nidulans* led to the identification of more than 100 compounds with >100-fold increase or decrease in abundance; among those were novel fellutamides, emericellamides, and aspercryptins [157,158]. However, it is not yet clear whether the observed effects were directly triggered by hyperacetylation of histones, transcription factors, and/or specific key enzymes of SM biosynthesis pathways (e.g., [165]), or whether SM was indirectly induced by stress response due to growth restrictions during RpdA depletion. Attempts to reverse the metabolomic effects by RpdA OE would be one plausible approach to exclude the latter possibility. However, although promoters such as *xylP*p are excellently suited for the expression of classical KDACs far beyond the wild-type level [166], no such data have been published for *Aspergillus* so far. This strongly suggests that, according to the observations described for HosA [103], sole OE of RpdA without an increase in corresponding complex partners will not lead to significant transcriptional effects on the fungal metabolome. Interestingly, however, RpdA OE in the necrotrophic mold *B. cinerea* results in a dramatically reduced transcription of many genes. Among those are members of the *Boa* and *Bot* gene clusters that regulate the production of the metabolites botrydial and botcinic acid, most likely as a result of RpdA-driven H3 hypoacetylation on distinct promoter regions [87]. In addition to these virulence-related molecules, stress-induced genes were significantly affected by RpdA OE in *B. cinerea* strains, and in fact, stress response is another known stimulus for increased SM production in many molds.

### 4.7. Microbial Interactions and the Induction of SMs: Are KDACs Involved in a Biochemical Warfare for Resources?

In addition to radicals, temperature, and other environmental sources, stress of fungi can also be triggered by rival microbes. Bacteria such as *Streptomyces, Pseudomonas*, and *Bacillus* might considerably contribute to fungal stress symptoms, either directly by competing for limited resources in the same habitat or indirectly by excreting specific metabolites. To combat such invaders, fungi keep distance from potential rivals by the production of repellents, such as antibiotic metabolites or toxins. On the other hand, also symbiotic interactions between microbes occur, and fungal compounds are only induced under intimate physical interaction with algae or prokaryotes. In some rare cases, “fungal” toxins are even produced by bacteria within fungal cells [167]. Anyhow, cocultivation of filamentous fungi with other microbes has become another promising approach to activate the production of otherwise silent SMs of fungi. Already more than one decade ago, interesting data on bacterial–fungal interaction triggering SM production came from the Brakhage group, when repressed metabolites such as orsellinic acid and lecanoric acid of *A. nidulans* were induced through interaction with *Streptomyces rapamycinicus* [168]. Interestingly, the same metabolites were also significantly increased in *Aspergillus* KDAC mutants and in wild-type strains grown under the KDAC inhibitor TSA [103], pointing to a link between KDACs and the effects on SM production in mixed *Aspergillus/Streptomyces* cultures. In this context, it is important to mention that several *Streptomyces* species are actually natural producers of TSA. A direct connection between *Streptomyces* and inhibition of *Aspergillus* KDACs in cocultures, however, could not yet be proven. Therefore, it can only be speculated that in cocultures, inhibition of fungal KDACs by bacterial metabolites might add to the induction of certain fungal metabolites, whereas production of others, such as PN, is silenced. In such a scenario, metabolites such as TSA might be considered a bacterial response against antibiotics such as PN in a chemical warfare of microbes for limited resources [103]. In the case of TSA, this is even more conceivable, as this metabolite also possesses direct antifungal properties [126], most likely by an inhibition of essential fungal KDACs such as RpdA [17,86]. Although mixed fermentation of bacteria and fungi is another possible approach to increase the chemodiversity of SM production in laboratories, the overall impact of microbial communications goes far beyond a possible impairment of fungal KDACs. The highly complex interplay taking place during microbial coexistence, however, is covered in detail in several other pertinent reviews (e.g., [23,169]).

## 5. Concluding Remarks and Perspectives

The identification of the first classical KDACs from *A. nidulans* [147] was the kickoff for functional investigations on this enzyme family in a large number of filamentous fungi. These investigations soon unveiled considerable structural differences between orthologs of higher eukaryotes, yeast, and molds. Additionally, even within filamentous fungi, a remarkable functional diversity was observed, pointing to a heavy rewiring of classical KDACs during fungal evolution. Therefore, the role of individual KDACs for both virulence traits and production of small bioactive compounds in a specific fungus can hardly be predicted.

Nevertheless, individual members of this versatile enzyme family were identified as common regulators of SM production at least across some species, suggesting the use of KDACIs for mining novel SMs. However, opposing regulatory effects of different KDAC classes impedes the unreflected use of these inhibitors, and the negative impact of KDACIs on fungal vitality sometimes complicates the interpretation of regulatory effects of KDACs on SM production.

**Figure 3:**
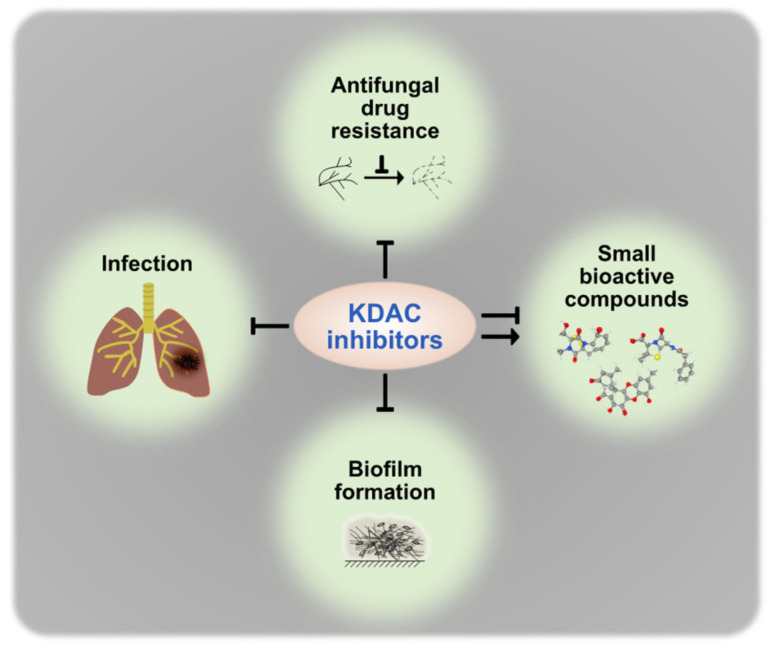
Effects of KDACIs on virulence-specific traits and SM production of fungi.

On the other hand, this vital role and the fact that new generations of potent KDACIs are under evaluation as anticancer drugs in clinical trials promoted the idea to use these compounds also for antifungal treatment. Indeed, a bunch of studies link KDAC depletion to virulence defects (Table 1). Notwithstanding their specific impact in distinct species, KDACs and their complex partners can be considered today as interesting new antifungal target candidates. The prospects for success of KDACIs as antifungal molecules are all the more promising, as classical azole therapy in combination with inhibition of KDACs exhibits additive effects and attenuates fungal resistance to azole derivatives [123]. The effects of KDACIs on fungal virulence and the production of bioactive compounds are summarized in Figure 3.

Although intolerance or serious side effects of KDACIs are not considered a major problem in anticancer therapies [170], antifungal KDACIs should be as specific and effective as possible. The fact that several selective inhibitors have already been successfully developed against human KDACs [171] illustrates that despite the high conservation of the region to be targeted by conventional KDACIs, formidable specificity can be achieved by a sophisticated inhibitor design. Currently, several projects are dealing with in silico analysis of specific domains or features of fungal KDACs, which hold out the prospect of a structure-guided drug design of novel inhibitors with highly targeted properties.

Prior to the evaluation of a large number of novel KDACIs in animal systems, ethical and economic reasons require a previous efficacy testing in vitro. For this purpose, purification of specific KDAC complexes from pathogenic fungi and mammalian cells will be one interesting possibility to evaluate the substrate specificity of newly designed molecules in enzymatic assays (e.g., [67]). Moreover, checkerboard assays can be used to test the antifungal activity of the molecules, either as single drugs or in combination with already-established antifungals under axenic growth conditions. At the very end of a successful prescreening process, however, suitability has to be confirmed in vivo. Within the last years, several nonmammalian models have been successfully adapted for their use in medical mycology. These invertebrate systems can be perfectly applied for in vivo evaluation of novel drugs, enabling high throughput within a short time at a low cost prior to the final efficacy testing in classical murine virulence models [172,173,174,175].

From today’s perspective, however, it is hard to predict whether the search for fungal-specific inhibitors will be successful within the next few years. Anyway, findings about toxicity profiles of KDACIs do not even exclude an administration of pan-inhibitors in adapted therapy regimens against life-threatening fungal infections.

Finally, together with KATs and sirtuins, classical KDACs must be appreciated as decisive players maintaining a delicate balance of protein acetylation crucial for SM production, virulence, and even the survival of many fungal species. In line with this, disturbance of this equilibrium, either on the KAT or on the KDAC side, has deleterious effects, particularly for fungal pathogens. 

## Figures and Tables

**Table 1:** Virulence phenotypes of fungal pathogen lysine deacetylase (KDAC) mutants. Names of KDACs and their FungiDB [99] or NCBI accession number, followed by the respective genetic modification, virulence phenotype, and host organism, are shown. Human- (top) and mainly plant-pathogenic (bottom) species are separated by a border line.

Pathogen	Rpd3 Homolog	Hos2 Homolog	Hda1 Homolog
*Aspergillus* *fumigatus*	RpdA (Afu2g03390)/ KD ^1^/av ^2^/mouse [89]	HosA (Afu2g03810) n.d. ^3^	HdaA (Afu5g01980)/ KO/fv/mouse [95]
*Candida* *albicans*	Rpd31 (C3_07000W_A) KO/av/mouse [70]	Hos2 (C3_00780W_A) n.d.	Hda1 (CR_02050C_A) n.d.
*Cryptococcus* *neoformans*	Rpd302 (CNAG_05096) KO/att/mouse [34] Rpd303 (CNAG_05276) KO/att/mouse [34] Rpd304 (CNAG_05690) KO/att/mouse [92]	Hos2 (CNAG_05563) KO/att/mouse [34]	Hda1 (CNAG_01563) KO/av/mouse [94] KO/att/mouse [34]
*Beauveria* *bassiana*	Rpd3 (EJP69682.1) KO/att/*Galleria* [84]	Hos2 (XP_008603650.1) KO/att/*Galleria* [85]	Hda1/Clr3 (EJP66596.1) n.d.
*Botrytis* *cinerea*	Rpd3 (Bcin05g02590) OE/att/tomato [64]	Hos2 (Bcin01g03610) n.d.	Hda1 (Bcin15g02130) n.d.
*Cochliobolus* *carbonum*	Hdc2 (AAK35180.1) n.d.	Hdc1 (AAL56814.1) KO/att/maize [49]	AAP95014.1 n.d.
*Fusarium* *graminearum*	Rpd3/Hda3 (FGRAMPH1_01G01959) KO/av/wheat [77]	Hdf1/Hda2 (FGRAMPH1_01G03337) KO/att/wheat, corn [76]	Hdf2/Hda1 (FGRAMPH1_01G15009) n.d.
*Ustilago* *maydis*	Hda1 (U6MAG_02065) KO/fv/maize [32] KO/fv/maize [83] Hda2 (UMAG_11308) KO/fv/maize [83]	Hos2 (UMAG_11828) KO/att/maize [83]	Hda1/Clr3 (UMAG_02102) KO/att/maize [83]
*Magnaporthe* *oryzae*	Rpd3 (MGG_05857) OE/av/rice, barley [82]	Hos2 (MGG_01633) KO/av/rice, barley [63] KO/att/rice [79]	Hda1 (MGG_01076) KO/att/rice [80]

^1^ KD—knockdown, KO—knockout, OE—overexpression, ^2^ av—avirulent, att—attenuated, fv—fully virulent, ^3^ n.d.—virulence phenotype not determined.

## Data Availability

Not applicable.

## Supplementary Materials

The following are available online at https://www.mdpi.com/article/10.3390/genes12101470/s1: Table S1: Nomenclature of fungal KDACs in different fungal species.
