# Supplementary material for: Fungal Lysine Deacetylases in Virulence, Resistance, and Production of Small Bioactive Compounds
Source: Genes (Basel). 2021 Sep 23;12(10):1470. doi: 10.3390/genes12101470 (PMC8535771; doi:10.3390/genes12101470)
Supplement: Supplementary file 1 [file genes-12-01470-s001.zip › genes-1377269-supplementary.pdf]

genes MDPI

Table S1. Nomenclature of fungal KDACs in different fungal species.

Organism	Class 1			Class2
Saccharomyces cerevisiae	Rpd3 (YNL330C)	Hos2 (YGL194C)	Hda1 (YNL021W)	Hos3 (YPL116W)
Schizosaccharomyces pombe	Clr6 (SPBC36.05c)	Hos2/Phd1 (SPAC3G9.07c)	Clr3 (SPBC800.03)	No ortholog
Candida albicans	Rpd3 (CR_02760C_A) Rpd31 (C3_07000W_A)	Hos2 (C3_00780W_A)	Hda1 (CR_02050C_A)	Hos3 (C4_02300W_A)
Aspergillus nidulans	RpdA (AN4493)	HosA (AN3806)	HdaA (AN8042)	HosB (AN7019)
Aspergillus fumigatus	RpdA (Afu2g03390)	HosA (Afu2g03810)	HdaA (Afu5g01980)	HosB (Afu4g04290)
Fusarium graminearum	Hda3 (FGRAMPH1_01G01959)	Hdf1/Hda2 (FGRAMPH1_01G03337)	HDF2Hda1 (FGRAMPH1_01G15009)	FGRAMPH1_01G18387
Magnaporthe oryzae	Rpd3 (MGG_05857)	Hos2 (MGG_01633)	Hda1 (MGG_01076)	Hos3 (MGG_06043)
Botrytis cinerea	Rpd3 (Bcin05g02590)	Hos2 (Bcin01g03610)	Hda1 (Bcin15g02130)	Bcin12g01310
Beauveria bassiana	Rpd3 (EJP69682.1)	XP_008603650.1	Hda1/Clr3 (EJP66596.1)	XP_008600884.1
Ustilago maydis	Hda1 (UMAG_02065) UMAG_11308	Hos2 (UMAG_11828)	UMAG_02102	UMAG_10914
Cochliobolus carbonum	Hdc2 (AAK35180.1)	Hdc1 (AAL56814.1)	AAP95014.1	AAP94999.1
Cryptococcus neoformans	Rpd304 (CNAG_05690) Rpd301 (CNAG_01699) Rpd302 (CNAG_05096) Rpd303 (CNAG_05276)	Hos2 (CNAG_05563)	Hda1 (CNAG_01563)	Hos3 (CNAG_00660)
